# ERGA-BGE Genome of the viperine water snake ( *Natrix maura*): a key species for evolutionary insights of freshwater snakes

**DOI:** 10.12688/openreseurope.20863.1

**Published:** 2025-08-04

**Authors:** Salvador Carranza, Daniel Fernández-Guiberteau, Laura Blasón, Sergi Tulloch, Rita Monteiro, Astrid Böhne, Rosa Fernández, Nuria Escudero, Laura Aguilera, Marta Gut, Tyler S Alioto, Francisco Câmara Ferreira, Jèssica Gómez-Garrido, Fernando Cruz, Swati Sinha, Leanne Haggerty, Fergal Martin, Tom Brown

**Affiliations:** 1 Institute of Evolutionary Biology (IBE, CSIC-Universitat Pompeu Fabra), Barcelona, Spain; 2 CREAC, Centre de Recerca i Educació Ambiental de Calafell (GRENP-Ajuntament de Calafell), Tarragona, Spain; 3 Leibniz Institute for the Analysis of Biodiversity Change, Museum Koenig Bonn, Bonn, Germany; 4 Centro Nacional de Análisis Genómico (CNAG), Barcelona, Spain; 5 Universitat de Barcelona (UB), Barcelona, Spain; 6 European Molecular Biology Laboratory, European Bioinformatics Institute, Cambridge, UK; 7 Leibniz Institute for Zoo and Wildlife Research, Berlin, Germany; 8 Berlin Center for Genomics in Biodiversity Research (BeGenDiv), Berlin, Germany

**Keywords:** Natrix maura, genome assembly, European Reference Genome Atlas, Biodiversity Genomics Europe, Earth Biogenome Project, viperine water snake

## Abstract

The reference genome of the freshwater snake, *Natrix maura,* offers a crucial resource for uncovering the genetic basis of adaptability to freshwater environments, while providing insights into hybridization and gene flow within the genus *Natrix*. A total of 18 contiguous chromosomal pseudomolecules were assembled from the genome sequence, corresponding to the 16 autosomes and 2 sex chromosomes. This chromosome-level assembly encompasses 1.7 Gb, composed of 284 contigs and 209 scaffolds, with contig and scaffold N50 values of 36.5 Mb and 174.4 Mb, respectively.

## Introduction

*Natrix maura*, a member of the Colubridae family, is a medium-sized, moderately robust freshwater snake that typically grows to just under one meter in length. It has a well-defined triangular head, a relatively thick body, and a short tail. The snake often features a distinctive dorsal zigzag stripe running along its body to the tip of the tail, which, along with its morphological and behavioral traits, can lead to confusion with viper species found in the western Mediterranean ( Speybroeck *et al.*, 2016). This is an example of Batesian mimicry, where a harmless species imitates the appearance and actions of a dangerous one to avoid predation ( Santos, 2021). *N. maura* is widely distributed across the western Mediterranean region, ranging from Liguria in Italy, through central and southern France, across the entire Iberian Peninsula, and into the Mediterranean-climate zones of northwestern Africa, including Morocco, Tunisia, and Algeria. Introduced to Sardinia, Menorca, Mallorca and Corsica ( Speybroeck *et al.*, 2016).

*N. maura* is currently classified as ‘Least Concern’ on the IUCN Red List ( Mateo Miras *et al.*, 2009) in view of its wide distribution, presumed large population, and because it is unlikely to be declining fast enough to qualify for listing in a more threatened category.

*N. maura* thrives in aquatic environments such as rivers, lakes, and wetlands, particularly in warm, Mediterranean habitats ( Speybroeck *et al.*, 2016). These environments provide ideal conditions for the snake, where it hunts for prey such as amphibians and small fish. Additionally, *N. maura* plays an important ecological role as prey for larger predators, supporting the overall biodiversity and health of these ecosystems, contributing to the balance of species within its natural habitats ( Santos, 2021).

Developing a high-quality reference genome for *N. maura* is essential for advancing our understanding of its unique genetic adaptations to freshwater environments. This genomic resource will not only enhance our knowledge of its physiological and ecological traits but also provide valuable insights into the evolutionary origins of venom in snakes. Additionally, it will offer broader implications for research on snake evolution and adaptation, contributing to our understanding of biodiversity and their ecological roles ( Schöneberg *et al.*, 2023).

*N. maura* may host parasites like trematodes, cestodes, and nematodes. These parasites are primarily found in the intestines but can also affect other parts of the digestive and respiratory systems, such as the pharynx, cloaca, trachea, and lungs ( Santos, 2021). The snake's varied diet and habitat contribute to its high parasite diversity, with some parasites being acquired from common prey like amphibians ( Santos, 2021).

The generation of this reference resource was coordinated by the European Reference Genome Atlas (ERGA) initiative’s Biodiversity Genomics Europe (BGE) project, supporting ERGA’s aims of promoting transnational cooperation to promote advances in the application of genomics technologies to protect and restore biodiversity ( Mazzoni *et al.*, 2023). This species falls within the regional reach of the Catalan Initiative for the Earth BioGenome Project (CBP), which is linked to ERGA ( Corominas *et al.*, 2024).

## Materials & methods

ERGA's sequencing strategy includes Oxford Nanopore Technology (ONT) and/or Pacific Biosciences (PacBio) for long-read sequencing, along with Hi-C sequencing for chromosomal architecture, Illumina Paired-End (PE) for polishing (i.e. recommended for ONT-only assemblies), and RNA sequencing for transcriptomic profiling, to facilitate genome assembly and annotation.

### Sample and sampling information

**Figure 1.**
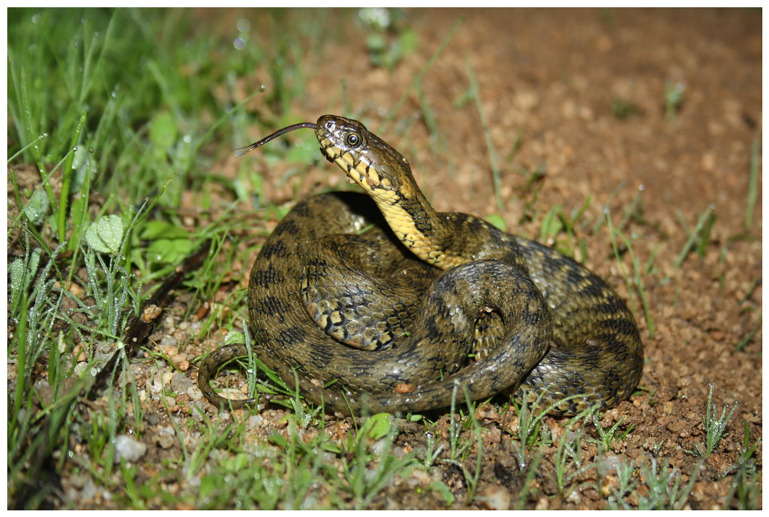
Example image of *Natrix Maura*. Image of *Natrix maura* taken by Daniel Fernández-Guiberteau. Further voucher images of the here sequenced specimen are available at the BioImage Archive under accession number SAMEA114541135: https://www.ebi.ac.uk/biostudies/BioImages/studies/S-BIAD1012

On 13th October 2023, an adult, female specimen of *Natrix maura* ( Figure 1) was sampled by Salvador Carranza and Daniel Fernández-Guiberteau. The species identification via morphology based on Santos 2021 and barcoding was confirmed by Salvador Carranza, Daniel Fernández-Guiberteau and Laura Blasón. The specimen was collected by hand from the Siurana River, Poboleda, Tarragona, Spain. Sampling was performed under permit number SF/0179/23 issued to Salvador Carranza by the Generalitat de Catalunya. Euthanasia was performed by first administering anesthetic agents (alfaxalone [Alfaxan, 10 mg/kg intramuscular]), and once the animal was fully anesthetized, the spinal cord was severed at the neck to ensure death. The specimen's tissues were snap-frozen in liquid nitrogen immediately after harvesting and stored at -80°C until DNA extraction.

### Vouchering information

Physical reference materials for the here sequenced specimen have been deposited in Museo Nacional de Ciencias Naturales (MNCN) https://www.mncn.csic.es, under the accession numbers MNCN-ADN-151759 & MNCN-ADN-151760.

Frozen reference tissue material of muscle tissue is available from the same individual at the MNCN under the voucher ID MNCN52208.

Images of the here sequenced specimen are available at the BioImage Archive under the accession SAMEA114541135: https://www.ebi.ac.uk/biostudies/BioImages/studies/S-BIAD1012

## Data availability

### Genetic information

The estimated genome size, based on direct measurements retrieved from Genomes on a Tree ( Challis *et al.*, 2023), is 2.3 Gb, while the estimation based on reads kmer profiling is 1.6 Gb. This is a diploid genome with a haploid number of 17 chromosomes (2n=34), including ZW sex chromosomes in female individuals ( De Smet, 1978).

### DNA/RNA processing

DNA was extracted from kidney tissue using the Blood & Cell Culture DNA Midi Kit (Qiagen) following the manufacturer’s instructions. DNA quantification was performed using a Qubit dsDNA BR Assay Kit (Thermo Fisher Scientific), and DNA integrity was assessed using a Genomic DNA 165 Kb Kit (Agilent) on the Femto Pulse system (Agilent). The DNA was stored at +4°C until used.

RNA was extracted using an RNeasy Mini Kit (Qiagen) according to the manufacturer’s instructions. RNA was extracted from four different specimen parts: ovary, liver, pancreas and lung. RNA quantification was performed using the Qubit RNA BR kit and RNA integrity was assessed using a Bioanalyzer 2100 system RNA 6000 Nano Kit (Agilent). The RNA was stored at -80°C until used.

### Library preparation and sequencing

For long-read whole genome sequencing, a library was prepared using the SQK-LSK114 Kit (Oxford Nanopore Technologies, ONT), which was then sequenced on a PromethION 24 A Series instrument (ONT). A short-read whole genome sequencing library was prepared using the KAPA Hyper Prep Kit (Roche). A Hi-C library was prepared from liver and lung using the ARIMA High Coverage Hi-C Kit (ARIMA), followed by the KAPA Hyper Prep Kit for Illumina sequencing (Roche). The RNA libraries were prepared using the KAPA mRNA Hyper prep kit (Roche). All the short-read libraries were sequenced on a NovaSeq 6000 instrument (Illumina).

In total, 52x Oxford Nanopore, 88x Illumina WGS shotgun, and 163x HiC data were sequenced to generate the assembly.

### Genome assembly methods

The genome was assembled using the CNAG CLAWS pipeline v2.2.0 ( Gomez-Garrido, 2024). Briefly, reads were preprocessed for quality and length using Trim Galore v0.6.7 and Filtlong v0.2.1, and initial contigs were assembled using NextDenovo v2.5.0, followed by polishing of the assembled contigs using HyPo v1.0.3, removal of retained haplotigs using purge-dups v1.2.6 and scaffolding with YaHS v1.2a. Finally, assembled scaffolds were curated via manual inspection using Pretext v0.2.5 with the Rapid Curation Toolkit ( https://gitlab.com/wtsi-grit/rapid-curation) to remove any false joins and incorporate any sequences not automatically scaffolded into their respective locations in the chromosomal pseudomolecules (or super-scaffolds). The blobtoolkit nextflow pipeline v0.6.0 ( https://pipelines.tol.sanger.ac.uk/blobtoolkit/0.6.0/usage) confirmed the absence of contaminants. Finally, the mitochondrial genome was assembled as a single circular contig of 21,237 bp using the FOAM pipeline v0.5 ( https://github.com/cnag-aat/FOAM) and included in the released assembly (GCA_964659585.1). Summary analysis of the released assembly was performed using the ERGA-BGE Genome Report ASM Galaxy workflow ( De Panis, 2024b).

### Genome annotation methods

A gene set was generated using the Ensembl Gene Annotation system ( Aken *et al.*, 2016), primarily by aligning publicly available short-read RNA-seq data from BioSamples: SAMEA114541140, SAMEA114541142, SAMEA114541145, and SAMEA114541148 to the genome. Gaps in the annotation were filled via protein-to-genome alignments of a select set of vertebrate proteins from UniProt ( The UniProt Consortium, 2019), which had experimental evidence at the protein or transcript level. At each locus, data were aggregated and consolidated, prioritising models derived from RNA-seq data, resulting in a final set of gene models and associated non-redundant transcript sets. To distinguish true isoforms from fragments, the likelihood of each open reading frame (ORF) was evaluated against known vertebrate proteins. Low-quality transcript models, such as those showing evidence of fragmented ORFs, were removed. In cases where RNA-seq data were fragmented or absent, homology data were prioritised, favouring longer transcripts with strong intron support from short-read data. The resulting gene models were classified into three categories: protein-coding, pseudogene, and long non-coding. Models with hits to known proteins and few structural abnormalities were classified as protein-coding. Models with hits to known proteins but displaying abnormalities, such as the absence of a start codon, non-canonical splicing, unusually small intron structures (<75 bp), or excessive repeat coverage, were reclassified as pseudogenes. Single-exon models with a corresponding multi-exon copy elsewhere in the genome were classified as processed (retrotransposed) pseudogenes. Models that did not fit any of the previously described categories did not overlap protein-coding genes, and were constructed from transcriptomic data were considered potential lncRNAs. Potential lncRNAs were further filtered to remove single-exon loci due to their unreliability. Putative miRNAs were predicted by performing a BLAST search of miRBase ( Kozomara *et al.*, 2019) against the genome, followed by RNAfold analysis ( Gruber *et al.*, 2008). Other small non-coding loci were identified by scanning the genome with Rfam ( Kalvari *et al.*, 2018) and passing the results through Infernal ( Nawrocki & Eddy, 2013). Summary analysis of the released annotation was performed using the ERGA-BGE Genome Report ANNOT Galaxy workflow ( De Panis, 2024a)

## Results

### Genome assembly

**Figure 2.**
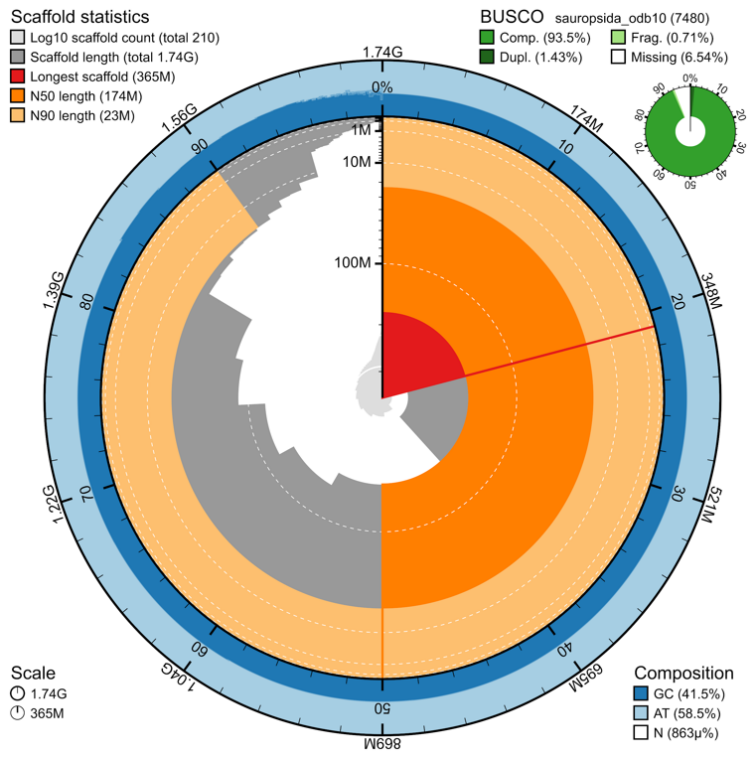
Snail plot summary of assembly statistics. The main plot is divided into 1,000 size-ordered bins around the circumference, with each bin representing 0.1% of the 1,738,090,269 bp assembly including the mitochondrial genome. The distribution of sequence lengths is shown in dark grey, with the plot radius scaled to the longest sequence present in the assembly (364,621,453 bp, shown in red). Orange and pale-orange arcs show the scaffold N50 and N90 sequence lengths (174,378,910 and 23,029,681 bp), respectively. The pale grey spiral shows the cumulative sequence count on a log-scale, with white scale lines showing successive orders of magnitude. The blue and pale-blue area around the outside of the plot shows the distribution of GC, AT, and N percentages in the same bins as the inner plot. A summary of complete, fragmented, duplicated, and missing BUSCO genes found in the assembled genome from the sauropsida database (odb10) is shown on the top right.

**Figure 3.**
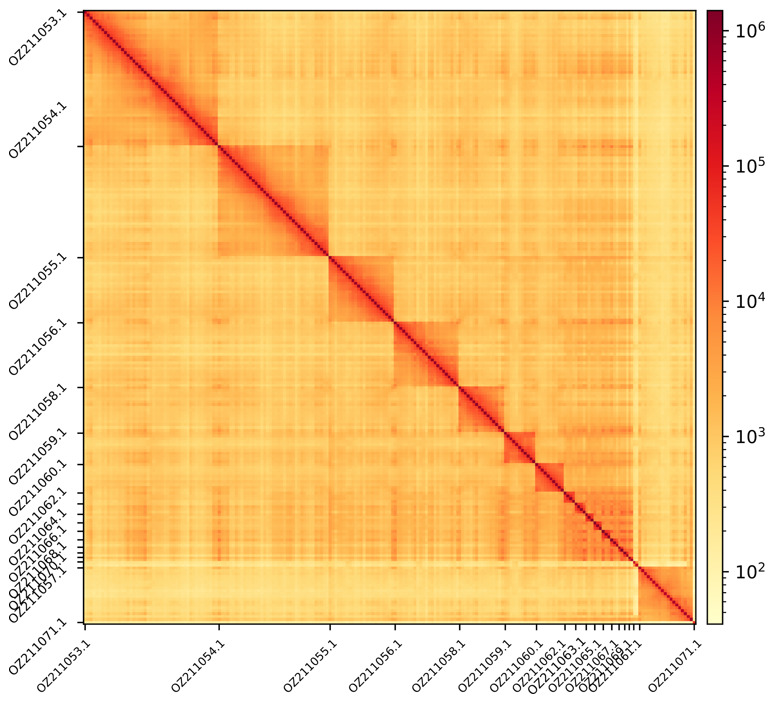
Hi-C contact map showing spatial interactions between regions of the genome. The diagonal corresponds to intra-chromosomal contacts, depicting chromosome boundaries. The frequency of contacts is shown on a logarithmic heatmap scale. Hi-C matrix bins were merged into a 150 kb bin size for plotting. Due to space constraints on the axes, only the GenBank names of the chromosomes are shown, with alternating names displayed for the micro-chromosomes on the x- and y-axes.

The genome assembly has a total length of 1,738,090,269 bp in 210 scaffolds including the mitogenome ( Figure 2 and Figure 3), with a GC content of 41.45%. It features a contig N50 of 36,526,383 bp (L50=13) and a scaffold N50 of 174,378,910 bp (L50=4). There are 75 gaps, totaling 15 kb in cumulative size. The single-copy gene content analysis using the sauropsida_odb10 database with BUSCO v5.5.0 resulted in 93.4% completeness (92.0% single and 1.4% duplicated). 91.35% of reads k-mers were present in the assembly and the assembly has a base accuracy Quality Value (QV) of 46.28 as calculated by Merqury.

### Genome annotation

The genome annotation consists of 17,402 protein-coding genes with an associated 51,854 transcripts, in addition to 27 pseudogenes and 1,491 non-coding RNA genes of various types ( Table 1). Using the longest isoform per transcript, the single-copy gene content analysis using the sauropsida_odb10 database with BUSCO v5.5.0 resulted in 93.4% completeness. Using the OMAmer Episquamata database for OMArk resulted in 95.85% completeness and 97.59% consistency ( Table 2).

**Table 1.** Statistics from assembled gene models.

	No. genes	No. transcripts	Mean gene length (bp)	No. single-exon genes	Mean exons per transcript
**Protein-coding**	17,402	51,854	41,684	491	15.2
**Pseudogenes**	27	27	7,337	2	6.7
**lncRNA**	981	1,663	21,796	88	3.6
**snRNA**	162	162	124	162	1.0
**snoRNA**	197	197	116	197	1.0
**rRNA**	73	73	610	73	1.0
**miRNA**	52	52	86	52	1.0
**scRNA**	15	15	194	15	1.0
**Other non-coding**	11	11	183	11	1.0

*Combined categories show the range of the mean values

**Table 2.** Annotation completeness and consistency scores calculated by BUSCO run in protein mode (Sauropsida_odb10) and OMArk (Episquamata).

	Complete	Singular	Duplicated	Fragmented	Missing
**BUSCO**	93.4%	91.7%	1.7%	0.9%	5.7%
**OMArk**	95.85%	93.78%	2.06%	-	4.15%
	Consistent	Inconsistent	Contaminants	Unknown	
**OMArk**	97.59%	1.57%	0.00%	0.84%	

## Data Availability

*Natrix maura* and the related genomic study were assigned to Tree of Life ID (ToLID) 'rNatMau1' and all sample, sequence, and assembly information are available under the umbrella BioProject PRJEB77787. The sample information is available at the following BioSample accessions: SAMEA114541135, SAMEA114541139, SAMEA114541140, SAMEA114541142, SAMEA114541148. The genome assembly is accessible from ENA under accession number GCA_964659585.1 and the annotated genome is available through the Ensembl ERGA-BGE project page ( https://projects.ensembl.org/erga-bge/) and Ensembl Genome data & annotation site ( https://beta.ensembl.org/). Sequencing data produced as part of this project are available from ENA at the following BioProject: PRJEB77785. Documentation related to the genome assembly and curation can be found in the ERGA Assembly Report (EAR) document available at https://github.com/ERGA-consortium/EARs/tree/main/Assembly_Reports/Natrix_maura/rNatMau1. Further details and data about the project are hosted on the ERGA portal at https://portal.erga-biodiversity.eu/data_portal/8585.
