# Supplementary material for: ERGA-BGE Genome of the viperine water snake ( Natrix maura): a key species for evolutionary insights of freshwater snakes
Source: Open Res Eur. 2025 Aug 4;5:215. [Version 1] doi: 10.12688/openreseurope.20863.1 (PMC12444268; doi:10.12688/openreseurope.20863.1)
Supplement: Peer review history

# Reviewer report: Reviewer response for version 1

**DOI:** 10.21956/openreseurope.22571.r59863

**Published:** 2025-09-18

**Authors:** Zexian Zhu

**Affiliations:** 1 Zhejiang University School of Medicine, Hangzhou, China

General comment:

 The authors present a high-quality, chromosome-level reference genome of Natrix maura, accompanied by a robust annotation. This genome will serve as an important resource for evolutionary, ecological, and conservation studies. The sequencing and assembly strategies follow standard protocols, and the results demonstrate good contiguity and completeness. I commend the authors for generating such a valuable dataset and genome report.

 A few aspects would benefit from clarification and additional detail to improve reproducibility and reader understanding.

 Detailed comments

1. The introduction emphasizes mimicry and distribution but less on the phylogenomic and comparative significance of this genome. It would be useful to explicitly connect N. maura with ongoing debates in snake evolution (e.g., sex chromosome evolution, repeated hybridization in Natrix, or genome size variation in colubrids)
2. The assembly metrics are excellent. However, the presentation is quite heavy. A concise comparative table (with other snake genomes’ statistics) would make it easier to read.
3. Figures 2 and 3 (snail plot and Hi-C map) are clear, but additional annotation of chromosome identity (e.g., the sex chromosomes) would add value. Even if preliminary, indicating which scaffolds correspond to sex chromosomes would enhance interpretability.
4. Assembly quality control. BUSCO, Merqury QV, and k-mer completeness are reported in the results, but the corresponding methods are missing. Please add details (e.g., software versions, parameters) in the Methods.

Minor suggestions:

1. Missing noun after ‘52x Oxford Nanopore’; also ‘data were sequenced” is inaccurate.’ It could be like ‘In total, sequencing yielded 52× coverage from Oxford Nanopore, 88× from Illumina WGS, and 163× from Hi-C libraries for the assembly.’
2. Words like ‘Likelihood’ in ‘…To distinguish true isoforms from fragments, the likelihood of each open reading frame (ORF) was evaluated against known vertebrate proteins…evidence of fragmented ORFs, were removed.’ is vague. It could be like ‘…To distinguish…, each ORF was compared against known…, and those with low-confidence (specify the threshold here, like frameshift or a premature stop codon?) were removed.
3. Missing a comma before the second ‘did’ in ‘…Models that did not fit any of the previously described categories did not overlap protein-coding genes, and were constructed from transcriptomic data were considered potential lncRNAs…’

Are sufficient details of methods and materials provided to allow replication by others?

Partly

Is the rationale for creating the dataset(s) clearly described?

Yes

Are the datasets clearly presented in a useable and accessible format?

Yes

Are the protocols appropriate and is the work technically sound?

Yes

Reviewer Expertise:

Genomic

I confirm that I have read this submission and believe that I have an appropriate level of expertise to confirm that it is of an acceptable scientific standard.

# Reviewer report: Reviewer response for version 1

**DOI:** 10.21956/openreseurope.22571.r58763

**Published:** 2025-09-02

**Authors:** Yannis Schoneberg

**Affiliations:** 1 Biogeography, Universitat Trier Biogeographie, Trier, Rhineland-Palatinate, Germany

The aim of this study was to generate a reference genome for Natrix maura. The authors clearly show that they succeeded in generating a high quality, chromosome level genome and a high quality annotation. Therefore, this genome will be a valuable resource for future studies on this species, including but not limited to evolution and conservation of this species. The methods are state of the art and mostly described in high detail. However, I found a few sections where clarity could be improved and details are missing.

 I congratulate the authors for generating such a valuable high quality genome resource and genome report.

 Best whishes

 Yannis Schöneberg

 #Detailed Comments:

 Unfortunately, it was not able to get a version with line numbers, even after inquiry with the editor. Therefore, I try to describe which text sections I mean.

 ## Comment 1

 "The species identification via morphology based on Santos 2021 and barcoding was confirmed by Salvador Carranza, Daniel Fernández-Guiberteau and Laura Blasón."

 I think the citation was not properly inserted.

 ## Comment 2

 "The species identification via morphology based on Santos 2021 and barcoding was confirmed by Salvador Carranza, Daniel Fernández-Guiberteau and Laura Blasón."

 It would be good to give a bit more details on how the barcoding was performed. Which marker sequence was used? Which Primers were used and how was it sequenced and how was the species identity confirmed?

 ## Comment 3

 "Data availability" (below Vouchering information)

 This header seems misplaced in the methods section.

 ## Comment 4

 "In total, 52x Oxford Nanopore, 88x Illumina WGS shotgun, and 163x HiC data were sequenced to generate the assembly"

 It would be good to speak of expected base coverage here, otherwise there might be room for misunderstandings.

 ## Comment 5

 Paragraph "Genome assembly methods"

 In this section, many citations for the programs are missing (e.g. TrimGalore or YaHS) and some existing ones are inadequate (e.g. the one of blobtoolkit). This should be revised.

 ## Comment 6

 "To distinguish true isoforms from fragments, the likelihood of each open reading frame (ORF) was evaluated against known vertebrate proteins. Low-quality transcript models, such as those showing evidence of fragmented ORFs, were removed."

 To me it's not entirely clear whether this is still part of the Ensemble Gene Annotation System, or if this was done by the authors manually. It would be good to have more information on how this was done.

 ## Comment 7

 "The genome assembly has a total length of 1,738,090,269 bp in 210 scaffolds including the mitogenome (Figure 2 and Figure 3), with a GC content of 41.45%. It features a contig N50 of 36,526,383 bp (L50=13) and a scaffold N50 of 174,378,910 bp (L50=4). There are 75 gaps, totaling 15 kb in cumulative size. The single-copy gene content analysis using the sauropsida_odb10 database with BUSCO v5.5.0 resulted in 93.4% completeness (92.0% single and 1.4% duplicated). 91.35% of reads k-mers were present in the assembly and the assembly has a base accuracy Quality Value (QV) of 46.28 as calculated by Merqury."

 The quality control methods are missing from the method section. They should be added e.g. in the "Genome assembly methods" section.

 ## Comment 8

 The genome annotation consists of 17,402 protein-coding genes with an associated 51,854 transcripts, in addition to 27 pseudogenes and 1,491 non-coding RNA genes of various types (Table 1). Using the longest isoform per transcript, the single-copy gene content analysis using the sauropsida_odb10 database with BUSCO v5.5.0 resulted in 93.4% completeness. Using the OMAmer Episquamata database for OMArk resulted in 95.85% completeness and 97.59% consistency (Table 2).

 The quality control methods are missing from the method section. They should be added e.g. in the "Genome annoation methods" section.

Are sufficient details of methods and materials provided to allow replication by others?

Partly

Is the rationale for creating the dataset(s) clearly described?

Yes

Are the datasets clearly presented in a useable and accessible format?

Yes

Are the protocols appropriate and is the work technically sound?

Yes

Reviewer Expertise:

Genome Assembly, Comparative Genomics, Phylogenetics

I confirm that I have read this submission and believe that I have an appropriate level of expertise to confirm that it is of an acceptable scientific standard.
